# Allometry of litter size in dog breeds

**DOI:** 10.1186/s13028-026-00862-9

**Published:** 2026-03-12

**Authors:** Klara Hanefeld Egeriis, Helle Friis Proschowsky, Bernt Guldbrandtsen

**Affiliations:** 1 Department of Veterinary and Animal Sciences, Animal Genetics & Breeding, University of Copenhagen, Grønnegårdsvej 3, DK-1870 Frederiksberg C, Denmark; 2 Department of Veterinary and Animal Sciences, Animal Welfare and Animal-Human Relations, University of Copenhagen, Grønnegårdsvej 8, DK- 1870 Frederiksberg C, Denmark

**Keywords:** Body weight, Brachycephaly, Chondrodysplasia, Litter size, Regression

## Abstract

Litter size varies among dog breeds and is related to body size. Generally, the relationship is described as small breeds having smaller litters, and larger breeds larger litters, though the trend does not extend to very large breeds. Allometric relationships are often log-log relationships. This study aims to investigate the relationship between body weight, body conformation, and litter size, with the hypothesis that: (1) body size and litter size are positively related with a power relationship and (2) breed-specific genetic characteristics, which impact body conformation, influence litter size. The present analysis addressed variation in litter size among 115 breeds and the effect of two breed-typical phenotypes, chondrodysplasia and brachycephaly. Log-log regression was used to investigate the relationship between litter size and body weight. A second-order relationship between log litter size and log body weight was observed. In conclusion, body size and litter size were strongly related. Brachycephaly had a significant negative effect on average litter size whereas litter size was unaffected by chondrodysplasia.

**Supplementary Information:**

The online version contains supplementary material available at 10.1186/s13028-026-00862-9.

## Findings

Several factors have been suggested to influence litter size in dogs including the size of the breed, the age of the bitch and the method of mating [1]. However, effects of chondrodysplasia and brachycephaly on litter size have not previously been investigated. Chondrodysplasia describes the short-legged phenotype in dog breeds like dachshunds, corgis and basset hounds. Brachycephaly describes the shortened skull and nasal bones in breeds like bulldog and pug. Information on factors affecting litter size among breeds can help extend the knowledge and may be useful to optimize breeding practices. The aim of this study is to fill this knowledge gap, including about the effects of chondrodysplasia and brachycephaly.

Litter size data for one year (August 2022 – July 2023) was provided from the Danish Kennel Club (DKC) in August 2023. The data included records for 258 breeds. Breeds with fewer than 10 litters recorded were removed, leaving 115 breeds in the full dataset.

Data on two breed-typical genetic characteristics (brachycephaly, chondrodysplasia) were obtained from Fédération Cynologique Internationale (FCI) [2]. Information on median weight in each breed was collected from DKC’s website, the dog encyclopaedia [3] in 2024. The distribution of median body weight in kg among breeds is presented in Additional file 1.

Including breeds that are closely related, as separate breeds is a potential source of pseudoreplication. A reduced dataset comprising 102 breeds was created using the following criteria: Breeds that share a common standard in FCI and where crossbreeding between variants such as fur type or size is allowed were identified, and only the most numerous breeds in each group were kept based on the registration figures provided by the DKC.

Additional file 2 show the breeds of the full and the reduced data set including the number of litters registered in the DKC from August 2022 to July 2023, average litter sizes, median body weight of each breed, and the breed characteristics chondrodysplasia or brachycephaly.

All statistical models were fitted in R using the lm function [4]. P-values < 0.05 were considered statistically significant. A log-log regression was performed of litter size on median weight. Presence of brachycephaly or chondrodysplasia of relevant breeds were included as covariates. To guard against the undue effect of related breeds, analyses were repeated in the reduced data set. Both first- and second-order relationships between variables were examined.

The full model analyzed was:

$$ \begin{aligned} {\mathrm{log}}_{2} \left( L \right)\, & = \,\beta _{1} {\mathrm{log}}_{2} \left( W \right) + \beta _{2} \left( {{\mathrm{log}}_{2} \left( W \right)} \right)^{2} \\ & + \beta _{C} C + \beta _{B} B + \beta _{0} + \epsilon, \\ \end{aligned} $$

where L was the average litter size in the breed, W was the median body weight, $C$ was an indicator variable taking the value 1 for chondrodysplastic breeds, and 0 otherwise, $B$ was an indicator variable taking the value 1 for brachycephalic breeds, and 0 otherwise, $ϵ$ was a random normal residual, and the various ${\beta}_{i}$ were fixed regression coefficients. ${\mathrm{l}\mathrm{o}\mathrm{g}}_{2}$ was the base-2 logarithm.

Models were gradually simplified by comparing complete models with reduced models using ANOVA.

The results of the regression analyses are shown in Table 1. Both the first and second order effects of the log of median body weight on litter size were highly significant (Additional files 3 and 4) Thus, the second order model provided a much better fit to the data than the first order model. The effect of brachycephaly was highly significant in both data sets (Additional files 4 and 5).

**Table 1:** Estimates of fixed effects from both datasets using first and second order models

Effect	Second order model		First order model
	Full data set estimate (se) *p* value	Reduced data set estimate (se) *p* value	Full data set estimate (se) *p* value
Number of breeds	115	102	115
Intercept	0.029 (0.232) *p* = 0.900	1.150 (0.268) *p* = 0.577	1.116 (0.114) *p* ≈ 0^***^
log_2_(W)	0.956 (0.129) *p* ≈ 0^***^	0.891 (0.145) *p* ≈ 0^***^	0.297 (0.027) *p* ≈ 0^***^
log_2_(W)^2^	-0.089 (0.017) *p* ≈ 0^***^	-0.079 (0.019) *p* ≈ 0^***^	NA
Brachycephaly	-0.418 (0.104) *p* ≈ 0^***^	-0.390 (0.104) *p* ≈ 0^***^	-0.402 (0.115) *p* ≈ 0^***^
Chondrodysplasia	0.135 (0.074) *p* = 0.068	0.069 (0.080) *p* = 0.389	0.147 (0.082) *p* = 0.076

The effect estimates are presented with standard error (se) and associated p-values. The second order model includes the first and second order effects of log_2_(W) while the first order model only evaluates linear relationships. The first order effect estimates of log_2_(W) are not directly comparable between the first and second order models. W represents the median body weight. Unavailable values are marked “NA”. Three stars (***) indicate parameters significantly different from zero

**Fig. 1:**
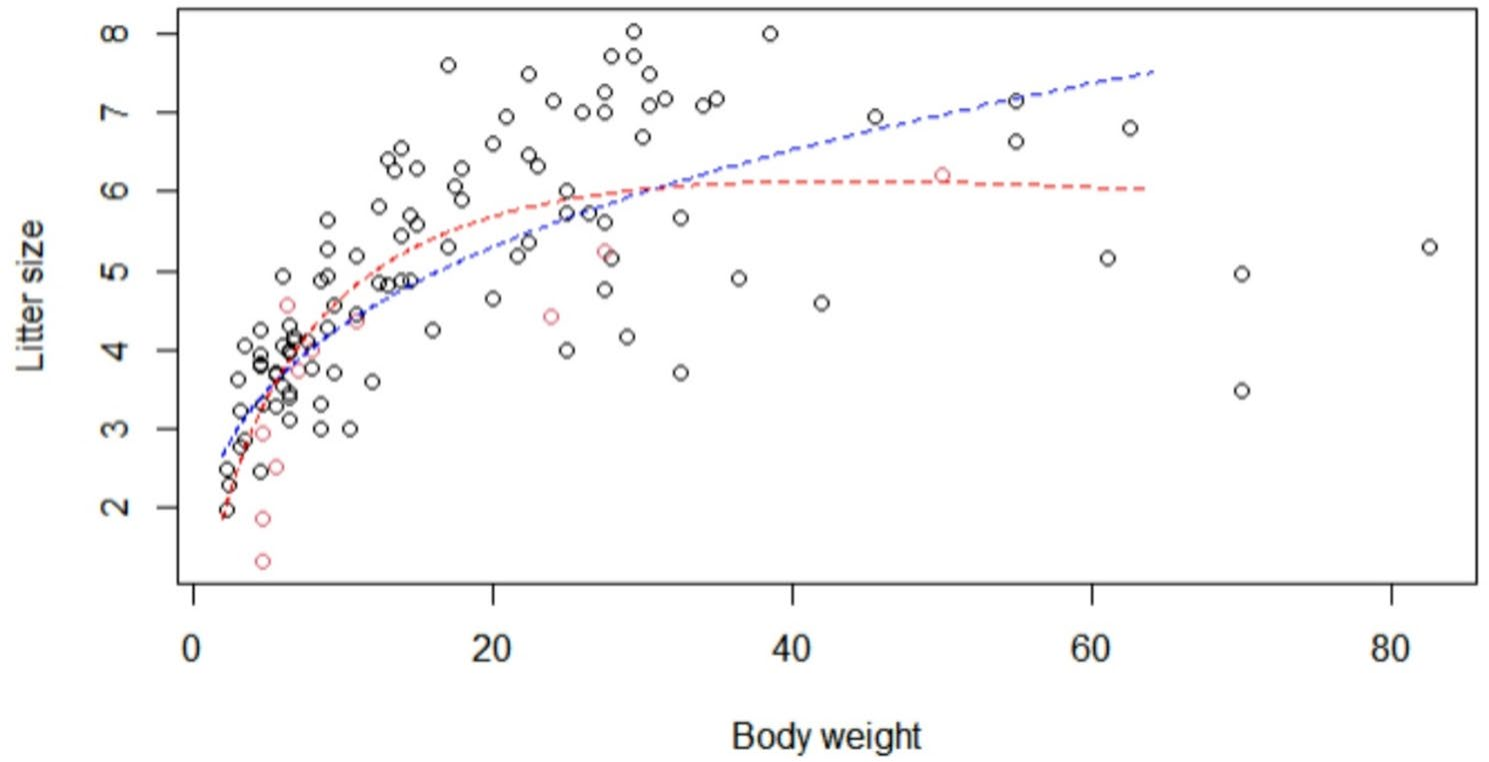
Shows the predicted values as function for first order model (blue line) andsecond order model (red line) with full dataset. Median body weight at the x-axis and litter sizeat the y-axis to visualize the relationship between those two factors. Black dots represent non-brachycephalic breeds, and red dots represent brachycephalic breeds. Both models seem todescribe data with the second order model to follow the dots a bit more perfectly

The data and fitted curves for non-brachycephalic breeds are shown in Fig. 1. The resulting prediction equations for the first and second order model for non-brachycephalic breeds are:

$${f}_{1}\left(W\right)=1.116+{\mathrm{log}}_{2}\left(W\right)\cdot0.297$$

$$ \begin{aligned} f_{2} \left( W \right)\, & = \,0.029 + {\mathrm{log}}_{2} \left( W \right)^{2} \cdot ( - 0.089) \\ & + {\mathrm{log}}_{2} \left( W \right) \cdot 0.956 \\ \end{aligned} $$

The first order equation can conveniently be rewritten as:

$$\hat{L} = 2.167 \cdot W^{{0.297}}$$

Here, $\hat{L}$ is the expected litters size for a non-brachycephalic breed with $W$ as median body weight.

In the second order model, the effect of brachycephaly was highly significant. If a breed is brachycephalic, the expected litter size is reduced by about $1-{2}^{-0.418}\approx25\mathrm{\%}$ compared to a non-brachycephalic breed with the same median body weight. The short-legged phenotype (chondrodysplasia) did not affect litter size.

The empirical cumulative distribution function of data approximately followed the cumulative distribution function for a normal distribution, indicating that the model provides a suitable fit to the data (Additional files 6 and 7).

A breed’s median body weight is a strong predictor for the average litter size in the breed. The parameter estimates were highly significantly different from zero.

Breeds with very high or very low body weight had smaller litter size than expected under a first order power law (Additional file 8). A second order model explained the data better. However, as seen in Fig. 1, for breeds with intermediate body weight, the first order prediction model did provide adequate predictions.

Previously, a regular linear regression had been applied [5], where outliers led to speculation of a non-linear relationship, Similarly, a multiple regression approach was applied [1] to model sizes of individual litters including breed as a random effect.

The results from the analyses conducted in our study support a curvilinear relationship. The largest and smallest breeds have smaller litters than predicted based on a first order power law (Additional file 6). Those deviations in litter size could be explained by biological constraints, where in small breeds, the relative size of the foetuses compared to the mother may limit the litter size and in giant breeds, the available space in the uterus may impose physical limitations on the number of puppies [1]. A second order model of the log-transformed data describes data including extreme breeds better than a simple linear regression does (results not shown).

For breeds with intermediate body weight, a doubling of the median body weight of a breed increases the expected litter size by a factor of ${2}^{0.297}-1\approx23\mathrm{\%}$. If only breeds with an average body weight of less than 40 kg were analysed, a more rapid increase of about 29% per doubling of body weight would be predicted (results not shown).

Repeating the analyses in the reduced data set did not alter conclusions. Hence, pseudoreplication was not a significant problem.

Litter size is reported by the breeders and may be underreported. For instance, the counting of stillborn offspring is not completely rigidly defined, meaning that the average litter size predicted may be somewhere between numbers born and numbers born alive. However, assuming the reporting issues occur uniformly among breeds, the predicted estimates of effects other than the intercept will not be strongly affected.

Brachycephalic breeds have a smaller average litter size than would be predicted based on their median body weight alone. Expected litter size was 25% smaller in brachycephalic breeds compared to non-brachycephalic breeds. This conclusion was not affected by removing closely related breeds. In particular, the detection of the effect is not caused by the inclusion of a few, closely related, brachycephalic breeds with small litters. However, no relation between the incidence of congenital anomalies (including brachycephaly) and litter size has previously been found [6].

In contrast, a breed being chondrodysplastic did not significantly affect average litter size in the breed.

Median body weight showed to be positively correlated with litter size. The relationship can be described by a second order power law. Brachycephalic breeds have lower average litter sizes than would be predicted based on their median body weight alone.

## Supplementary Information

Additional file 1. Present the distribution of median body weight in kg among breeds.

Additional file 2. Shows the breeds of the full and the reduced data set including the number of litters registered in the DKC from August 2022 to July 2023, average litter sizes, median body weight of each breed, and the breed characteristics chondrodysplasia or brachycephaly.

Additional file 3. Shows model fit for first order model, full dataset.

Additional file 4. Shows model fit for second order model, full dataset.

Additional file 5. Shows model fit for second order model, reduced dataset.

Additional file 6. Shows the empirical cumulative distribution function of residuals (red curve). Comparison of the observed cumulative distribution with the theoretical normal distribution (black points). Minor deviations from the normal distribution are observed, particularly in the tail regions where the empirical curve diverges from the red theoretical curve.

Additional file 7. Q-Q-plot over residuals represented in function f2(x). Compare the observed quantities with theoretical quantities for a normal distribution. The dots overall follow the line indicating normal distribution of data. Deviations in tail regions indicate some outliers that weigh more heavily.

Additional file 8. Shows the predicted values as function for first order model (blue line) and second order model (red line) with full dataset. log2 (median body weight) at the x-axis and log2 (litter size) at the y-axis to visualize the relationship between those two factors. Black dots represent non-brachycephalic breeds, and red dots represent brachycephalic breeds. Both models seem to describe data with the second order model to follow the dots a bit more perfectly.

## Data Availability

The datasets supporting the conclusions of this article are included within the article and its additional files.

## Declarations

### Prior publication

Few of the results have been presented in the bachelor thesis written by KHE. Data have not been published previously or used otherwise.
