# Supplementary figures and images for: Allometry of litter size in dog breeds
Source: Acta Vet Scand. 2026 Mar 12;68:20. doi: 10.1186/s13028-026-00862-9 (PMC13097871; doi:10.1186/s13028-026-00862-9)

**Additional file 1:** Present the distribution of median body weight in kg among breeds.

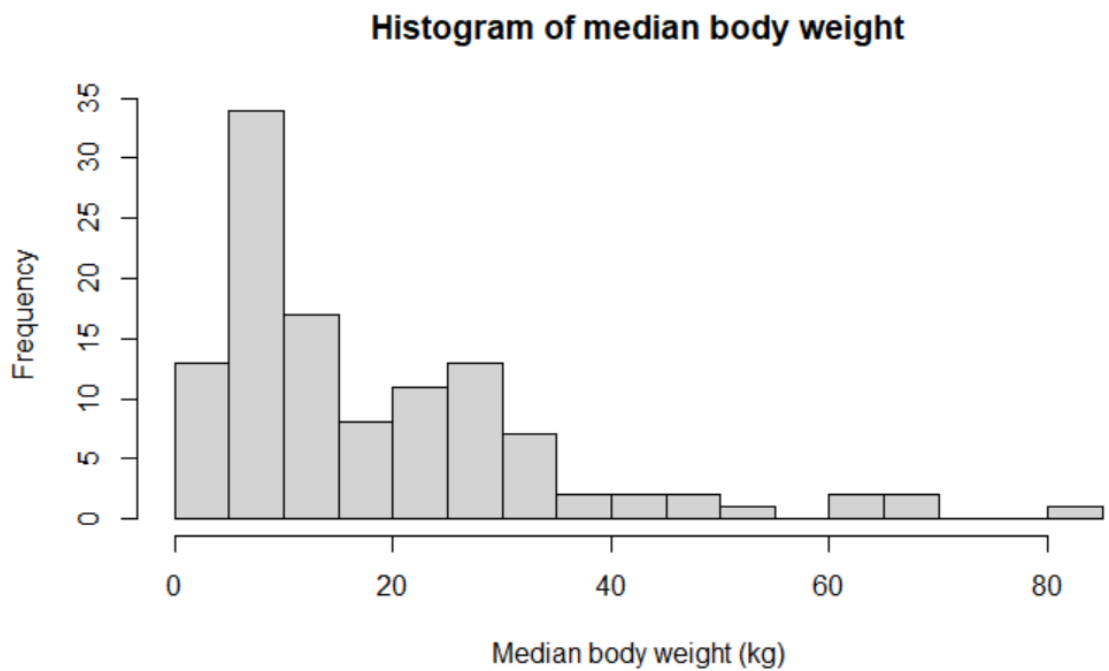

Supplement: Supplementary file 1 — Additional file 1. Present the distribution of median body weight in kg among breeds. [file 13028_2026_862_MOESM1_ESM.pdf]
