# Supplementary material for: Allometry of litter size in dog breeds
Source: Acta Vet Scand. 2026 Mar 12;68:20. doi: 10.1186/s13028-026-00862-9 (PMC13097871; doi:10.1186/s13028-026-00862-9)
Supplement: Supplementary file 2 — Additional file 2. Shows the breeds of the full and the reduced data set including the number of litters registered in the DKC from August 2022 to July 2023, average litter sizes, median body weight of each breed, and the breed characteristics chondrodysplasia or brachycephaly. [file 13028_2026_862_MOESM2_ESM.pdf]

Additional file 2: The 115 breeds of the full data set including the number of litters registered in the DKC from August 2022 to July 2023, average litter sizes, and median body weight of each breed in kg. Chondrodysplastic breeds are marked with a C, and brachycephalic breeds are marked with a B. The 13 breeds that were removed to produce the reduced dataset are written in regular font.

**Breed name**	**Number of litters**	**Average litter size**	**Median breed weight in kg**
**Alaskan malamute**	10	4.9	36.5
**American cocker spaniel**	14	3.6	12.0
**Australian shepherd**	64	7.2	24.0
**Basset fauve de Bretagne** [C]	10	5.3	17.0
**Basset hound** [C]	19	5.6	27.5
**Beagle**	38	5.2	11.0
**Belgian shepherd dog, Malinois**	14	7.7	28.0
Belgian shepherd dog, Tervueren	12	5.2	28.0
**Bernese mountain dog**	118	4.6	42.0
**Bichon frisé** [C]	31	4.3	4.5
**Border collie**	101	5.9	18.0
**Border terrier**	45	4.0	6.0
**Boston terrier** [B]	32	4.0	8.0
**Boxer** [B]	47	5.2	27.5
**Breton**	23	5.4	14.0
**Broholmer**	31	7.2	55.0
**Bull terrier**	13	4.2	29.0
**Bullmastiff**	14	6.2	50.0
**Cairn terrier** [C]	81	4.2	6.8
**Cavalier king Charles spaniel**	237	4.1	6.7
**Chihuahua, long-haired**	65	2.5	2.3
Chihuahua, smooth-haired	49	2.0	2.3
**Chinese crested dog**	16	3.7	5.5
**Chow chow**	27	4.0	25.0
**Collie, rough**	32	4.8	27.5
**Continental toy spaniel, papillon**	39	3.2	3.3
Continental toy spaniel, phalène	12	2.8	3.3
**Coton de tulear** [C]	258	3.3	4.7
**Dachshund, long-haired, miniature** [C]	29	3.8	4.5
Dachshund, long-haired, rabbit [C]	18	4.1	3.5
Dachshund, long-haired, standard [C]	17	4.9	9.0
Dachshund, wire-haired, miniature [C]	26	3.9	4.5
**Dachshund, wire-haired, standard** [C]	60	5.3	9.0
Dachhund, smooth-haired, miniature [C]	11	3.8	4.5
**Dachhund, smooth-haired, standard** [C]	19	5.6	9.0
**Dalmatian**	11	7.7	29.5
**Danish spitz**	17	6.3	15.0

**Danish-Swedish farm dog**	104	4.6	9.5
**Dobermann**	18	8.0	38.5
**Dutch kooikerhondje**	12	4.8	12.5
**Dutch schapendoes**	14	5.6	15.0
**English bulldog** [B]	34	4.4	23.9
**English cocker spaniel**	120	4.8	13.0
**English setter**	18	5.7	25.0
**English springer spaniel**	40	6.3	23.0
**Eurasier**	45	6.0	25.0
**Finnish lapponian dog**	60	4.7	20.0
**Flat coated retriever**	41	7.5	30.5
**Fox terrier (wire)**	20	4.1	7.7
**French bulldog** [B]	58	4.4	11.0
German shepherd dog, long-haired	48	3.7	32.5
**German shepherd dog, short-haired**	325	5.7	32.5
**German short-haired pointing dog**	37	7.0	27.5
**German wire-haired pointing dog**	75	8.1	29.5
**Golden retriever**	312	7.2	31.5
**Gordon setter**	11	7.3	27.5
**Great Dane**	22	6.8	62.5
**Griffon Belge** [B]	10	1.3	4.8
**Griffon Bruxellois** [B]	14	1.9	4.8
**Havanese** [C]	473	4.9	6.0
**Hungarian short-haired pointer (Vizsla)**	14	7.0	26.0
**Icelandic sheepdog**	45	4.9	14.5
**Irish setter**	10	7.6	17.0
**Irish softcoated wheaten terrier**	12	6.1	17.5
**Jack Russell terrier**	144	4.0	6.5
**Japanese spitz**	23	3.0	8.5
**Kromfohrländer**	11	5.8	12.5
**Labrador retriever**	629	6.7	30.0
**Lagotto romagnolo**	17	5.7	14.5
**Leonberger**	11	6.6	55.0
**Lhasa apso** [C]	32	4.3	6.5
**Little lion dog**	17	3.5	6.0
**Maltese** [C]	27	2.9	3.5
**Mastiff**	13	5.3	82.5
**Medium size spitz**	25	4.9	8.5
**Miniature American shepherd**	15	6.3	13.5
Miniature schnauzer, black	16	3.4	6.5
**Miniature schnauzer, black and silver**	30	3.4	6.5
Miniature schnauzer, peber and salt	18	3.4	6.5
**Newfoundland**	24	5.2	61.0
**Nova Scotia duck tolling retriever**	34	6.6	20.0
**Old Danish pointing dog**	22	7.1	30.5
**Parson Russell terrier**	12	4.0	6.5

**Pekingese [B, C]**	10	2.5	5.5
**Petit basset griffon vendéen [C]**	13	4.2	16.0
**Petit brabançon [B]**	27	2.9	4.8
**Pomeranian**	41	2.3	2.5
Poodle, medium size	40	4.3	9.0
**Poodle, miniature**	95	3.1	6.5
**Poodle, standard**	39	7.5	22.5
Poodle, toy	46	2.5	4.5
**Pug [B]**	43	3.7	7.0
**Rhodesian ridgeback**	18	7.1	34.0
**Rottweiler**	50	7.0	45.5
**Samoyed**	15	5.7	26.5
**Schipperke**	15	3.3	5.5
**Scottish terrier [C]**	13	3.7	9.5
**Shar pei**	17	5.4	22.5
**Shetland sheepdog**	106	3.8	8.0
**Shiba**	32	3.0	10.5
**Shih tzu [B, C]**	80	4.6	6.3
**Siberian husky**	10	5.2	21.8
**Small münsterländer**	64	7.0	21.0
**Spanish water dog**	17	6.3	18.0
**St. Bernard. long-haired**	23	5.0	70.0
St. Bernard. short-haired	17	3.5	70.0
**Stabijhoun**	28	6.5	22.5
**Staffordshire bull terrier**	195	4.9	14.0
**Tibetan spaniel**	67	3.7	5.5
**Welsh corgi cardigan [C]**	70	6.6	14.0
**Welsh corgi pembroke [C]**	22	4.5	11.0
**West highland white terrier [C]**	92	3.3	8.5
**Whippet**	30	6.4	13.0
**White Swiss shepherd dog**	17	7.2	35.0
**Yorkshire terrier [C]**	33	3.6	3.1
