# Supplementary material for: Allometry of litter size in dog breeds
Source: Acta Vet Scand. 2026 Mar 12;68:20. doi: 10.1186/s13028-026-00862-9 (PMC13097871; doi:10.1186/s13028-026-00862-9)
Supplement: Supplementary file 3 — Additional file 3. Shows model fit for first order model, full dataset. [file 13028_2026_862_MOESM3_ESM.pdf]

Additional file 3: Shows model fit for first order model, full dataset.

```
Call:
lm(formula = log2(litter.size) ~ log2(w) + chondrodystrof + brachycephal,
    data = data_full)

Residuals:
     Min       1Q   Median       3Q      Max 
-1.14186 -0.15995  0.04444  0.24702  0.59543 

Coefficients:
                  Estimate Std. Error t value Pr(>|t|)    
(Intercept)        1.11649    0.11419   9.778  < 2e-16 ***
log2(w)            0.29702    0.02707  10.974  < 2e-16 ***
chondrodystrofyes  0.14665    0.08193   1.790 0.076184 .  
brachycephalyes   -0.40166    0.11510  -3.490 0.000695 ***
---
Signif. codes:  0 ‘***’ 0.001 ‘**’ 0.01 ‘*’ 0.05 ‘.’ 0.1 ‘ ’ 1

Residual standard error: 0.3268 on 111 degrees of freedom
Multiple R-squared:  0.5842,	Adjusted R-squared:  0.573 
F-statistic: 51.99 on 3 and 111 DF,  p-value: < 2.2e-16
```
