# Supplementary material for: Allometry of litter size in dog breeds
Source: Acta Vet Scand. 2026 Mar 12;68:20. doi: 10.1186/s13028-026-00862-9 (PMC13097871; doi:10.1186/s13028-026-00862-9)
Supplement: Supplementary file 4 — Additional file 4. Shows model fit for second order model, full dataset. [file 13028_2026_862_MOESM4_ESM.pdf]

Additional file 4: Shows model fit for second order model, full dataset.

```
Call:
lm(formula = log2(litter.size) ~ I(log2(w)^2) + log2(w) + chondrodystrof +
    brachycephal, data = data_full)

Residuals:
     Min       1Q   Median       3Q      Max
-0.93321 -0.16534  0.03863  0.23317  0.53511

Coefficients:
                  Estimate Std. Error t value Pr(>|t|)
(Intercept)        0.02907    0.23217   0.125   0.9006
I(log2(w)^2)      -0.08866    0.01698  -5.222 8.44e-07 ***
log2(w)            0.95602    0.12852   7.439 2.38e-11 ***
chondrodystrofyes  0.13541    0.07370   1.837   0.0689 .
brachycephalyes   -0.41837    0.10355  -4.040 9.92e-05 ***
---
Signif. codes:  0 ‘***’ 0.001 ‘**’ 0.01 ‘*’ 0.05 ‘.’ 0.1 ‘ ’ 1

Residual standard error: 0.2938 on 110 degrees of freedom
Multiple R-squared:  0.6668,	Adjusted R-squared:  0.6547
F-statistic: 55.04 on 4 and 110 DF,  p-value: < 2.2e-16
```
