# Supplementary material for: Allometry of litter size in dog breeds
Source: Acta Vet Scand. 2026 Mar 12;68:20. doi: 10.1186/s13028-026-00862-9 (PMC13097871; doi:10.1186/s13028-026-00862-9)
Supplement: Supplementary file 5 — Additional file 5. Shows model fit for second order model, reduced dataset. [file 13028_2026_862_MOESM5_ESM.pdf]

Additional file 5: Shows model fit for second order model, reduced dataset.

```
Call:
lm(formula = log2(litter.size) ~ I(log2(w)^2) + log2(w) + chondrodystrof +
    brachycephal, data = data_red)

Residuals:
     Min       1Q   Median       3Q      Max
-0.98557 -0.15204  0.05527  0.21201  0.55612

Coefficients:
                  Estimate Std. Error t value Pr(>|t|)
(Intercept)        0.15007    0.26810   0.560 0.576936
I(log2(w)^2)      -0.07879    0.01880  -4.192 6.11e-05 ***
log2(w)            0.89058    0.14456   6.161 1.65e-08 ***
chondrodystrofyes  0.06884    0.07957   0.865 0.389111
brachycephalyes   -0.38978    0.10421  -3.741 0.000311 ***
---
Signif. codes:  0 ‘***’ 0.001 ‘**’ 0.01 ‘*’ 0.05 ‘.’ 0.1 ‘ ’ 1

Residual standard error: 0.2883 on 97 degrees of freedom
Multiple R-squared:  0.6678,    Adjusted R-squared:  0.6541
F-statistic: 48.76 on 4 and 97 DF,  p-value: < 2.2e-16
```
