# Supplementary material for: Allometry of litter size in dog breeds
Source: Acta Vet Scand. 2026 Mar 12;68:20. doi: 10.1186/s13028-026-00862-9 (PMC13097871; doi:10.1186/s13028-026-00862-9)

Additional file 6: Shows the empirical cumulative distribution function of residuals (red curve). Comparison of the observed cumulative distribution with the theoretical normal distribution (black points). Minor deviations from the normal distribution are observed, particularly in the tail regions where the empirical curve diverges from the red theoretical curve.

Cumulative distribution function of model residuals

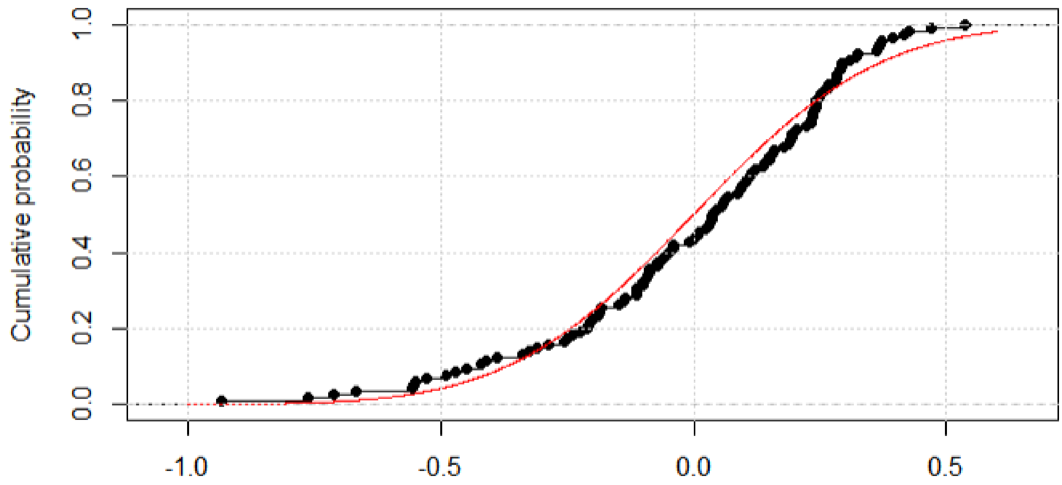

Supplement: Supplementary file 6 — Additional file 6. Shows the empirical cumulative distribution function of residuals (red curve). Comparison of the observed cumulative distribution with the theoretical normal distribution (black points). Minor deviations from the normal distribution are observed, particularly in the tail regions where the empirical curve diverges from the red theoretical curve. [file 13028_2026_862_MOESM6_ESM.pdf]
