# Supplementary material for: Allometry of litter size in dog breeds
Source: Acta Vet Scand. 2026 Mar 12;68:20. doi: 10.1186/s13028-026-00862-9 (PMC13097871; doi:10.1186/s13028-026-00862-9)

Additional file 7: Q-Q-plot over residuals represented in function f2(x). Compare the observed quantities with theoretical quantities for a normal distribution. The dots overall follow the line indicating normal distribution of data. Deviations in tail regions indicate some e outliers that weigh more heavily.

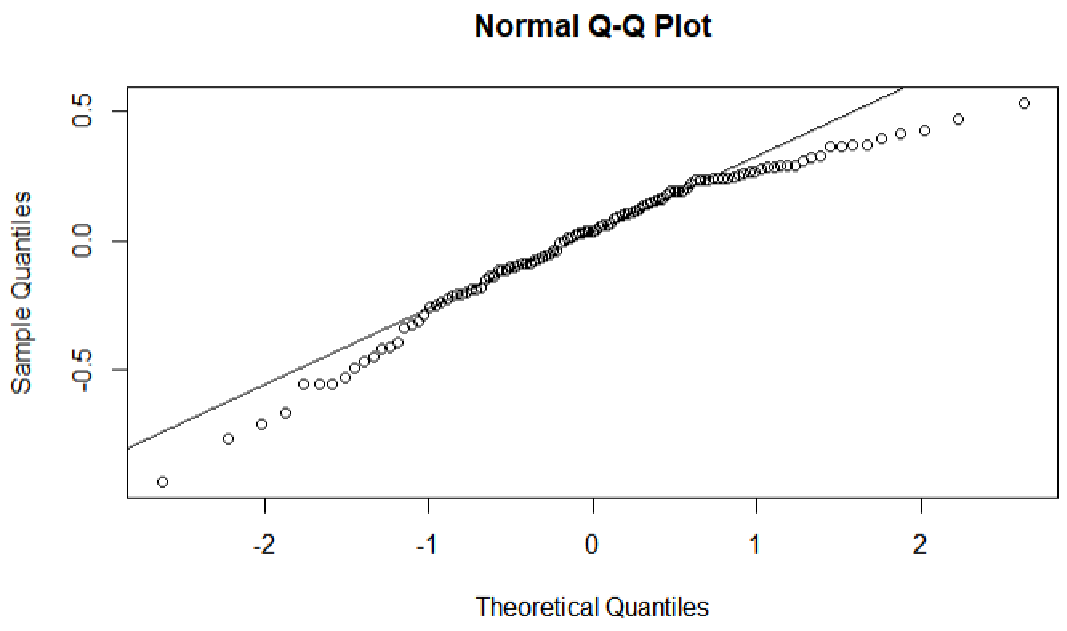

Supplement: Supplementary file 7 — Additional file 7. Q-Q-plot over residuals represented in function f2(x). Compare the observed quantities with theoretical quantities for a normal distribution. The dots overall follow the line indicating normal distribution of data. Deviations in tail regions indicate some outliers that weigh more heavily. [file 13028_2026_862_MOESM7_ESM.pdf]
