# Supplementary material for: Allometry of litter size in dog breeds
Source: Acta Vet Scand. 2026 Mar 12;68:20. doi: 10.1186/s13028-026-00862-9 (PMC13097871; doi:10.1186/s13028-026-00862-9)

Additional file 8: Shows the predicted values as function for first order model (blue line) and second order model (red line) with full dataset. log2 (median body weight) at the x-axis and log2 (litter size) at the y-axis to visualize the relationship between those two factors. Both models seem to describe data with the second order model to follow the dots a bit more perfectly.

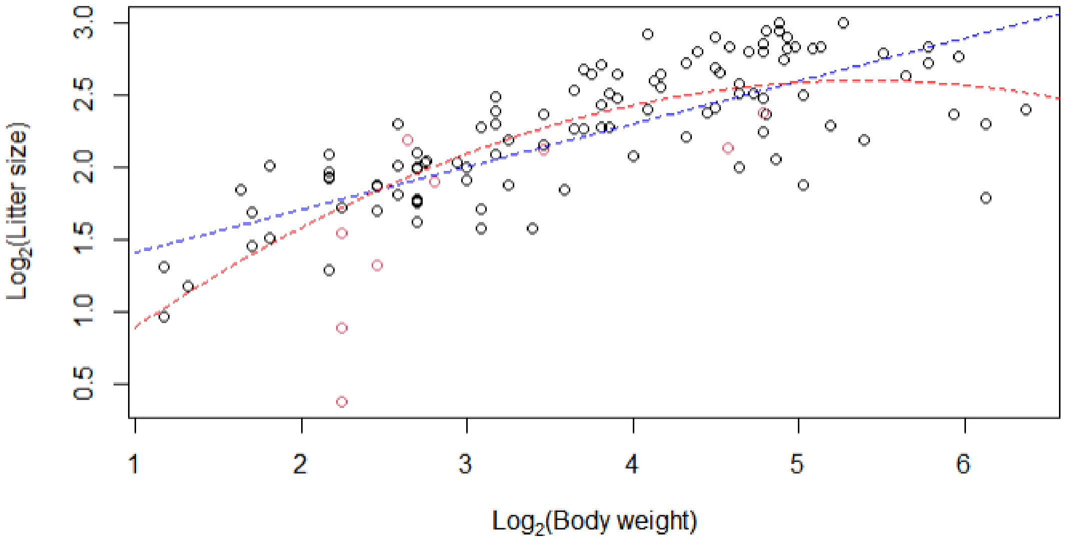

Supplement: Supplementary file 8 — Additional file 8. Shows the predicted values as function for first order model (blue line) and second order model (red line) with full dataset. log2 (median body weight) at the x-axis and log2 (litter size) at the y-axis to visualize the relationship between those two factors. Black dots represent non-brachycephalic breeds, and red dots represent brachycephalic breeds. Both models seem to describe data with the second order model to follow the dots a bit more perfectly. [file 13028_2026_862_MOESM8_ESM.pdf]
